# Abstract of Stenographer’s Report, U. S. V. M. A., 1895

**Published:** 1896-01

**Authors:** 


Secretary.

### ABSTRACT OF STENOGRAPHER'S REPORT, U. S. V. M. A., 1895.

THE thirty-second annual meeting was called to order at 10.30 A. M. by President Hoskins, in Horticultural Room, Capitol Building, Des Moines, Iowa. The President then introduced Honorable W. S. Richards, private secretary to Governor Jackson, who apologized for the Governor's absence from the State, and then, in well-chosen words, gave a hearty and generous welcome to all present. President Hoskins responded with sincere appreciation of the warm welcome accorded the Convention, and followed with his annual address.[1]

On roll-call, the following members responded to their names: Drs. Brainerd, Cary, F. H. P. Edwards, Faust, Gill, Harger, Harrison, H. G. Hoover, G. A. Johnson, C. P. Lyman, J. C. Meyer, Jr., McBirney, W. B. Niles, Osgood, A. F. Peters, T. B. Rayner, Salmon, Schwartzkopf, Stalker, S. Stewart, Trumbower, Wattles, Whitbeck, Williams.

Visitors: Drs. J. H. McLeod, Harold Sorby, William Drinkwater, S. H. Johnson, P. Macolm, H. G. Stover, C. J. Huerdey, G. M. Walrod, J. Miller, J. G. Parslow, G. A. Scott, N. R. Fullerton, J. B. Hollenbeck, J. A. Replogle, J. H. Gasson, W. H. Austin, C. A. Clinton, C. E. Garmen.

As delegates from Pennsylvania: J. C. Foelker and T. B. Rayner; Iowa: J. E. Brown; Massachusetts: F. H. Osgood; Missouri: G. T. Netherton. Representing the Iowa State Board of Health: Messrs. Carter, Dickinson, and Kennedy.

The Secretary then read the minutes of the meeting of 1894, which were approved.

The report of the *comitia minora* was then read and the following recommendations acted upon: That we accept the resignations of Drs. J. E. Cloud, J. H. Frinck, R. D. Eaton, Alexander Glass, and W. A. Hitchcock, which, on motion, was approved; that we expel from membership Drs. D. D. Lee and E. C. Beckett, of Boston, Mass., and G. J. Leo Hagenburger, of Brooklyn, for violation of the code of ethics, in unprofessional forms of advertising, in the case of Messrs. Lee and Beckett, and for the same reason and the placing upon the market of proprietary preparations, in the case of Dr. Hagenburger. Recommendation, on motion, adopted.

The following applicants, having complied with all the requirements, were favorably recommended for membership:

Ellis, Bird, Gray, John R. Hart, Killer, C. J. Marshall, Enoch H. Moore, Reagan, Joshua Miller, Arnold, Eagan, Emila Pouppert, L. W. Shipley, C. C. Cole, Edwin Hogg, C. B. McClelland, Sherrard, Charles Saunders, Fred. E. Pierce, Henry Shipley, C. M. Day, Thomas A. Bray, G. T. Netherton.; and, on motion, duly elected.

[1] See page 708, November, 1895.

The roll of delinquents was then read by the Secretary.

The President referred to the unprecedented amount on the books unpaid, and said the work of the Association the past year had been greatly curtailed owing to the unfortunate condition of the treasury.

He asked for information from any present as to any reason why action should not be taken on any of the names read. The *comitia minora* recommended that sixty days after notice by the Secretary to delinquents of the amounts against their names due the Association, remaining unpaid, that the same be placed in a collection agency's hands for collection. This measure was discussed by several members, and, on motion, adopted with but one dissenting voice. The Secretary was instructed to carry it into execution at once.

The President then referred to the very large number of deaths among our members during the past year, and, in referring to the deceased, appointed committees to draft suitable resolutions of our regard for these deceased ones:

Dr. Robert Leis—Drs. H. D. Gill, J. C. Meyer, Jr., Samuel S. Whitbeck; Dr. W. H. Paaren—Drs. D. E. Salmon, M. R. Trumbower, C. P. Lyman; Dr. W. Bryden—Drs. F. H. Osgood, R. H. Harrison, John Faust; Dr. J. F. Autenrieth—Drs. R. H. Harrison, S. J. J. Harger, W. B. Niles; Dr. W. D. Middleton—Drs. John Faust, James L. Robertson, W. J. Coates.

Dr. M. Stalker then asked permission of the Chair to say a few words, when, on behalf of the State Board of Health, he informed the members that their offices on this floor of the Capitol were open, and to which a generous welcome was extended to all; that the State Fair was now in progress at Des Moines, and that if the Association could find time to attend in a body, it would be a great pleasure to the Iowa Veterinarians in providing for the same.

Dr. Williams then gave notice in writing, signed by Dr. Osgood and himself, of a proposed amendment to the by-laws; changing the same in regard to the date of meeting, which now reads "on first Tuesday in September," to read, "at such time as the Executive Committee may elect," stating the object to be that advantageous rates might be obtained in conjunction with other organizations. The President directed its reference to the *comitia minora*, to be reported back at our next meeting.

Dr. C. P. Lyman then offered a resolution, duly signed, changing the present name of *comitia minora* to that of Executive Committee as a better and more intelligent designation for this body. This was ordered by the Chair to take the same course.

Dr. R. H. Harrison then offered the name of Dr. John Desmond-Warmambool, Australia, for honorary membership. It was referred to the *comitia minora* to be reported back in September, 1896.

Reports of committees being in order, that of Intelligence and Education was called for and read by Dr. C. A. Cary, Chairman, together with a report by Dr. W. B. Niles, read by the latter, and one from Dr. R. A. Archibald, read by Chairman Cary, entitled "How Shall the Practitioner Continue His Education?"

The several reports were then opened for discussion, and Dr. Salmon, taking the floor, objected to the direct allusion by Chairman Cary to certain colleges, and thought the author was too severe upon them, and that he placed too high a value on the courses at agricultural colleges.

Dr. Stalker said that it was rather a good plan to hold out a positive example of a school defective in its scope of teaching and then bring the others around this one for comparison. He felt there was great need in determining the qualifications first that a student should possess before becoming a veterinarian, and that the present demanded in students a higher literary training than ever before. He strongly favored a long curriculum in time, for it affords so much greater an opportunity to use even meagre facilities of teaching to so much greater advantage.

Dr. Schwarzkopf thought the Chairman was too severe in his criticisms of American veterinary colleges, and did not give them just credit for the good work accomplished under so many trying circumstances. He referred to the remarkable unity of action in the Association of Faculties, of the great triumphs already achieved, and how promising the future, and, further, that it was a time when every encouragement should be given those who were so deeply interested in this forward movement of the colleges.

The President then asked that all should remember that while the freest and fullest and broadest of discussion should follow all these committee reports, that it did not follow that these reports were indorsed by the Association; they were never considered in such a light, except where distinct resolutions or conclusions were specifically stated and submitted for such action.

Dr. C. P. Lyman in discussing the report referred to the very salutary effects in the past resulting from reports of this committee, and especially to the suggestion that has resulted in so strong and valued an organization as the Association of Faculties stands to-day. He thought greater good was to flow from a discussion of such a report of this kind from those not interested or directly connected with the teaching staffs of the schools, and from them new plans and suggestions would emanate that might receive the recommendation of the Association, and be considered in the Association of Faculties for the further advancement of the work being done by this body.

Dr. Osgood desired that a freer recognition be given to the work done by agricultural colleges, and considered them the best preparatory schools for veterinary colleges that we have.

Dr. Williams spoke of the worth of agricultural college training as a preparatory course, but felt that what most schools needed to-day was better education on more practical lines. He referred to the difficulty of arriving at a just estimate of the scope of teaching as laid down in the various college catalogues. He felt like strongly supporting the present great advances in many schools, and aiding them in better grading their courses and improving their faculties, leading to a more thorough practical education and training.

Dr. Harger felt that great good had already been obtained by agitation in this Association, and warmly indorsed the tendencies to increase its requirements for admission; he also felt that State legislation looking to the establishment of State Boards of Examiners, who would thus discern the progressive and thoroughly earnest schools, thus strengthening their hands and stimulating the laggard ones to better and more advanced efforts. He differed on one point with the Chairman, and strongly approved of the teachers in certain branches of a practical character to have private practices, from which so much valuable material may at times be garnered.

After corrections on misunderstood points by Drs. Schwarzkopf and Williams, the meeting adjourned until 2.15 P.M.

AFTERNOON SESSION.

On being called to order by the President, the report of the Committee on Diseases was called for, and Chairman Trumbower responded. All his colleagues had been enthusiastically at work, and the result was the most complete and voluminous ever presented to the Association. The report contained a very interesting communication from Prof. Liautard in France. The use of Pasteur's anthrax vaccine in New Jersey on some three thousand animals with very satisfactory results was reported.

Section B of the report, concerning anthrax, black leg, and tuberculosis, having been assigned to Dr. John Faust, was called for, and the latter responded, bringing out some salient points relative to tuberculosis and the value of preventive inoculation in anthrax; after which the meeting adjourned until 10 A.M. of the 11th.

(To be concluded.)

